# Effect of micro-computed tomography voxel size and segmentation method on trabecular bone microstructure measures in mice

**DOI:** 10.1016/j.bonr.2016.05.006

**Published:** 2016-05-27

**Authors:** Blaine A. Christiansen

**Affiliations:** UC Davis Medical Center, Department of Orthopaedic Surgery, 4635 2nd Ave, Suite 2000, Sacramento, CA 95817, USA

**Keywords:** Trabecular bone, Microstructure, Micro-computed tomography, Voxel size, Resolution, Segmentation

## Abstract

Micro-computed tomography (μCT) is currently the gold standard for determining trabecular bone microstructure in small animal models. Numerous parameters associated with scanning and evaluation of μCT scans can strongly affect morphologic results obtained from bone samples. However, the effect of these parameters on specific trabecular bone outcomes is not well understood. This study investigated the effect of μCT scanning with nominal voxel sizes between 6–30 μm on trabecular bone outcomes quantified in mouse vertebral body trabecular bone. Additionally, two methods for determining a global segmentation threshold were compared: based on qualitative assessment of 2D images, or based on quantitative assessment of image histograms. It was found that nominal voxel size had a strong effect on several commonly reported trabecular bone parameters, in particular connectivity density, trabecular thickness, and bone tissue mineral density. Additionally, the two segmentation methods provided similar trabecular bone outcomes for scans with small nominal voxel sizes, but considerably different outcomes for scans with larger voxel sizes. The Qualitatively Selected segmentation method more consistently estimated trabecular bone volume fraction (BV/TV) and trabecular thickness across different voxel sizes, but the Histogram segmentation method more consistently estimated trabecular number, trabecular separation, and structure model index. Altogether, these results suggest that high-resolution scans be used whenever possible to provide the most accurate estimation of trabecular bone microstructure, and that the limitations of accurately determining trabecular bone outcomes should be considered when selecting scan parameters and making conclusions about inter-group variance or between-group differences in studies of trabecular bone microstructure in small animals.

## Highlights

• This study investigated the effect of μCT scanning with voxel sizes between 6–30 μm on mouse trabecular bone measures.
• Two commonly used segmentation methods for determining a global segmentation threshold were compared.
• Voxel size strongly affected trabecular bone parameters including connectivity density and trabecular thickness.
• The segmentation methods yielded similar outcomes for scans with small voxel sizes, but not scans with large voxel sizes.
• Suggest that high-resolution scans should be used when possible to accurately estimate trabecular bone microstructure.

## 1 Introduction

Micro-computed tomography (μCT) is the gold standard for quantifying trabecular and cortical bone microarchitecture in small animal models (Bouxsein et al., 2010). MicroCT is able to directly measure trabecular bone architecture without having to rely on stereological models that were previously utilized for histological assessment of bone structure (Hildebrand et al., 1999, Weibel, 1980). However, there are numerous variables associated with the data acquisition, processing, and evaluation of μCT scans that can affect morphologic results obtained from bone samples. Bouxsein et al. published guidelines for μCT studies in small animal models (Bouxsein et al., 2010), which has helped to standardize the reporting of study parameters and results, however the effects of various scan parameters on the morphologic results obtained are not fully known.

The voxel size for a μCT scan can strongly affect trabecular or cortical bone results if the voxel size is not appropriately small compared to the dimensions of the structure being measured (Kim et al., 2004). Voxel size has a negligible effect for analysis of structures with relatively high thickness relative to the nominal voxel size (< 10:1). However, when analyzing small structures such as mouse trabeculae (20–70 μm), which have dimensions on the same order as the smallest voxel size of most commercially available μCT systems (1–10 μm), voxel size can have significant effects on the results (Muller et al., 1996). Ideally, the smallest voxel size (highest scan resolution) available would be used for all μCT scans. However, high-resolution scans are not always desirable since they require longer acquisition times and generate large data sets. Additionally, if μCT scans are performed on live animals *in vivo*, long scan times and higher radiation dose become important concerns.

Segmentation, the process of binarizing images to “bone” and “non-bone” is also an important process in μCT analysis that can strongly affect trabecular bone morphology results. Most studies of small animal trabecular bone utilize a “global threshold” which is applied to all samples in a study. However, the methods for selecting this threshold are not consistent between research groups, and are not always clearly communicated. Some studies utilize quantitative threshold selection, for example using the midpoint of the “bone” and “non-bone” peaks of the histogram of the local voxels of a sample (Dufresne, 1998). Regardless of the segmentation used, it is recommended to visually compare segmented and grayscale images to confirm that the segmentation is representative of the “physiologic” structure of the trabecular bone (Bouxsein et al., 2010).

This study investigated the effect of μCT voxel size on trabecular bone morphology indices quantified in mouse vertebral body trabecular bone. Additionally, two methods for determining segmentation threshold based on either qualitative assessment of 2D images, or quantitative assessment of image histograms were compared. Results from this study will help guide future studies of small animal trabecular bone using μCT, and will help researchers compare results from studies that used different voxel sizes.

## 2 Methods

**Fig. 1:**
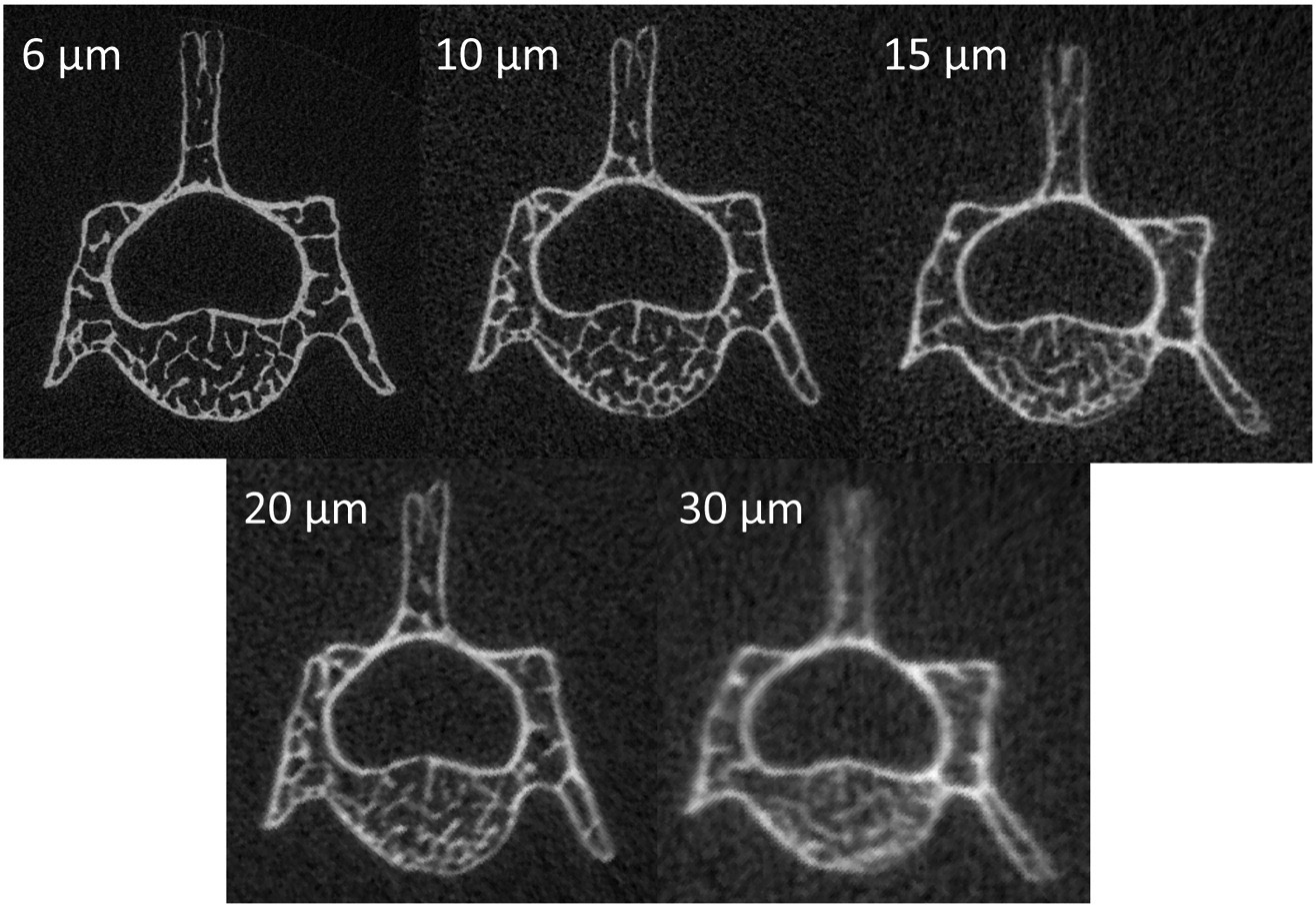
Raw (unsegmented) micro-computed tomography (μCT) images of the same mouse lumber vertebra scanned with nominal voxel sizes from 6–30 μm.

L5 vertebrae from six adult (12 week-old) male C57BL/6N mice (Harlan Sprague Dawley, Indianapolis, IN) were scanned using a commercially available micro-computed tomography system (SCANCO μCT 35, Brüttisellen, Switzerland) according to the guidelines for μCT analysis of rodent bone structure (Bouxsein et al., 2010): X-ray tube potential = 55 kVp, current = 114 μA, integration time = 900 ms, number of projections = 1000/180**°**. Serial scans were performed on the same bone samples with isotropic nominal voxel sizes of 6, 10, 15, 20, and 30 μm (Fig. 1). The trabecular region of the vertebral body (excluding posterior elements) was designated using manually drawn contours inside the cortical shell on two-dimensional transverse slices by a single experienced operator, encompassing the entire vertebral body enclosed by the growth plates.

**Fig. 2:**
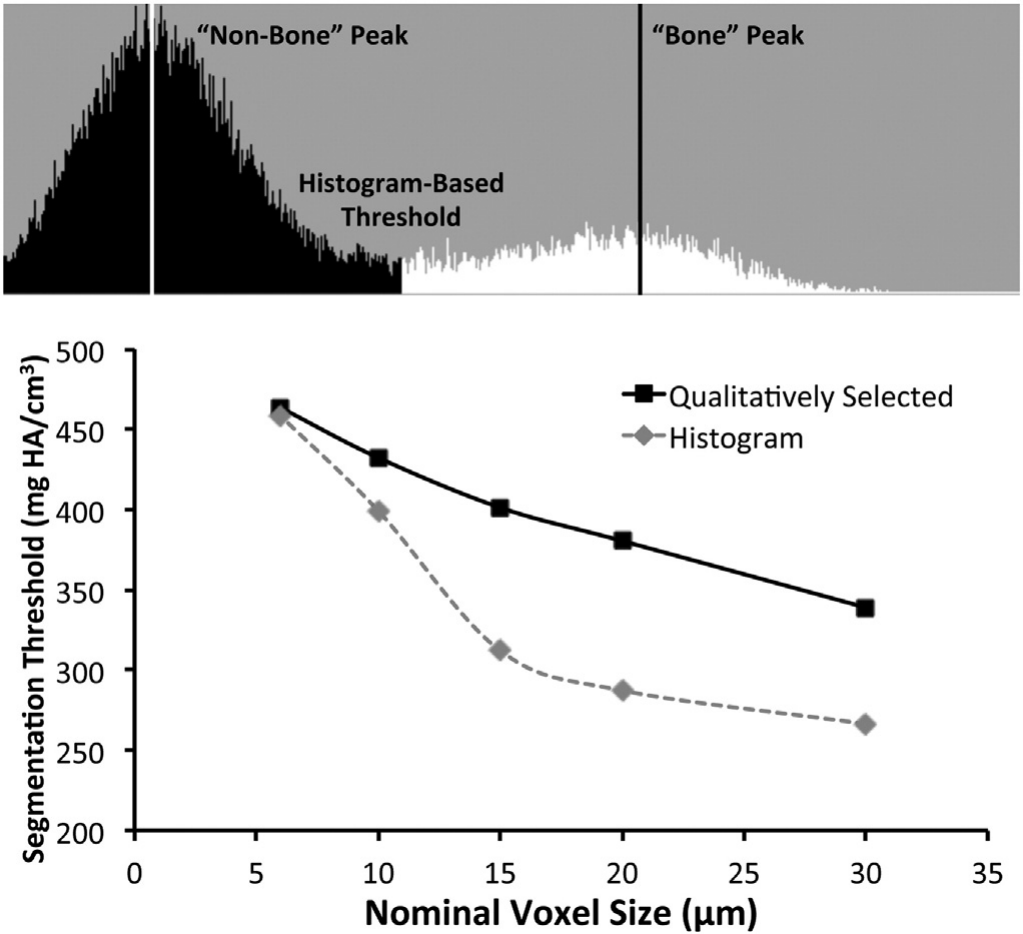
(Top) For the histogram-based segmentation method, the “bone” and “non-bone” histogram peaks were identified, and the midpoint between these peaks was selected as the global segmentation threshold (histogram from a 6 μm voxel size scan). (Bottom) For smaller voxel sizes (6–10 μm), the two segmentation methods selected similar thresholds (1–8% difference), while for larger voxel sizes (15–30 μm) the two methods selected considerably different thresholds (21–25% difference).

Segmentation threshold for image analysis was determined for scans of each voxel size using two methods. First, threshold was selected qualitatively by an experienced operator by comparing segmented trabecular bone to original grayscale images, with the goal of obtaining a physiologically accurate representation. Second, segmentation threshold was determined quantitatively from the histogram of the trabecular compartment as previously described (Dufresne, 1998). For this method the threshold was set at the midpoint between the “bone” and “non-bone” peaks of the histogram (Fig. 2).

Trabecular bone volume fraction (BV/TV), connectivity density (Conn.Dens), structure model index (SMI), trabecular thickness (Tb.Th), trabecular separation (Tb.Sp), trabecular number (Tb.N), bone tissue mineral density (Tissue BMD; mg HA/cm^3^ BV), and apparent mineral density (Apparent BMD; mg HA/cm^3^ TV) were directly measured using the manufacturer's 3-D analysis tools. All outcomes were compared using ANOVA to determine differences from “true” values (defined as values obtained for the 6 μm voxel size).

## 3 Results

“Qualitatively Selected threshold” based on subjective selection by an experience operator for physiologic representation, and “Histogram threshold” based on the voxel brightness histogram were both strongly dependent on scan voxel size (Fig. 2). For larger voxel sizes (15–30 μm) the two methods selected considerably different segmentation thresholds (21–25% difference), while for smaller voxel sizes (6–10 μm) the two methods selected comparable segmentation thresholds (1–8% difference).

**Fig. 3:**
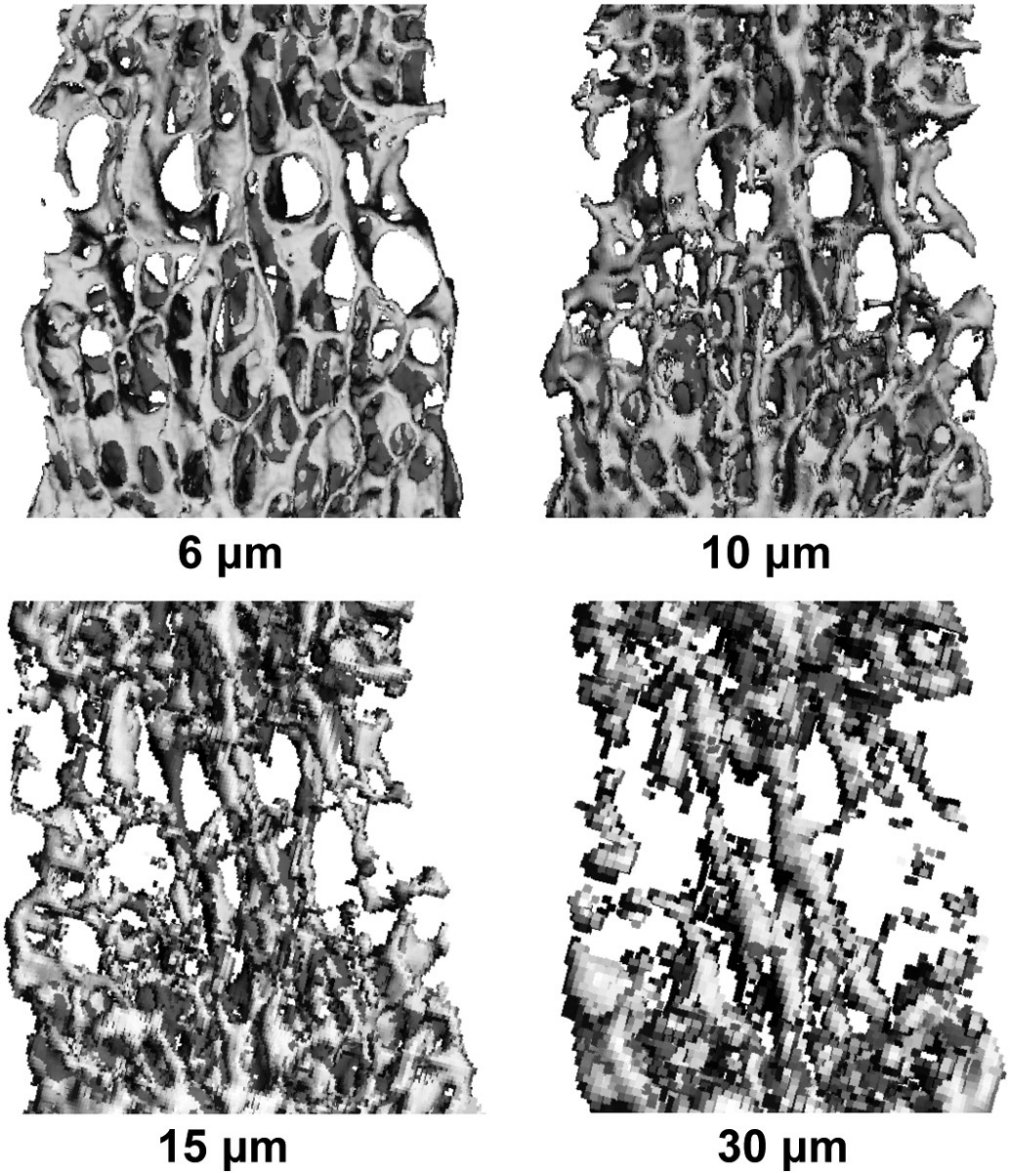
3D μCT reconstructions of the trabecular bone volume from the same lumbar vertebral body analyzed from scans with four different nominal voxel sizes. The Qualitatively Selected segmentation method was used for these images.

**Fig. 4:**
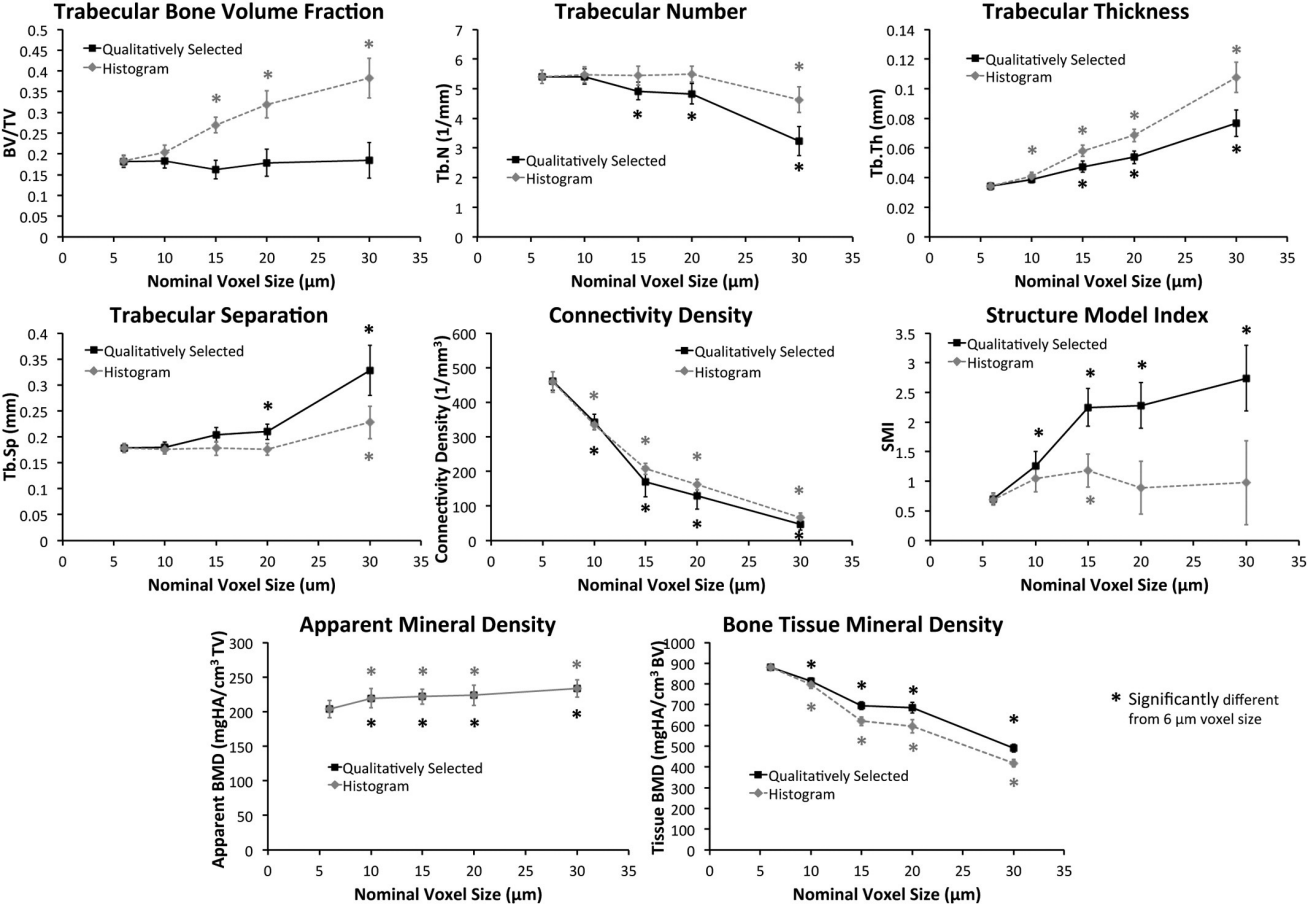
Graphs showing changes in commonly reported trabecular microstructure outcomes as a function of scan nominal voxel size using two different segmentation methods.

Nominal voxel size of scans had a strong effect on the resolution of trabecular microstructure (Fig. 3) and several of the calculated trabecular bone parameters (Fig. 4). Using the Qualitatively Selected segmentation method, trabecular bone volume fraction (BV/TV) was not different for any of the scan voxel sizes. However, other outcomes were significantly affected when using this segmentation method. For example, Conn.Dens decreased from 461.6 mm^− 3^ at 6 μm voxel size to 46.7 mm^− 3^ at 30 μm voxel size (− 90%), Tb.Th increased from 34.0 μm at 6 μm voxel size to 76.7 μm at 30 μm voxel size (+ 126%), and Tissue BMD decreased from 881.3 mg HA/cm^3^ at 6 μm voxel size to 490.8 mg HA/cm^3^ at 30 μm voxel size (− 44%).

Using the Histogram segmentation method, BV/TV was significantly increased 47–109% for voxel sizes from 15–30 μm relative to values obtained with 6 μm voxel size. Similarly, Conn.Dens decreased from 458.3 mm^− 3^ at 6 μm voxel size to 65.7 mm^− 3^ at 30 μm voxel size (− 86%), Tb·Th increased from 34.2 μm at 6 μm voxel size to 107.6 μm at 30 μm voxel size (+ 215%), and Tissue BMD decreased from 880.2 mg HA/cm^3^ at 6 μm voxel size to 418.6 mg HA/cm^3^ at 30 μm voxel size (− 52%). The Histogram segmentation method yielded more consistent results for Tb.N, Tb.Sp, and SMI than the Qualitatively Selected segmentation method, with no significant differences observed for these values for most voxel sizes compared to the 6 μm voxel size.

## 4 Discussion

This study investigated the effect of scan voxel size on trabecular bone morphology indices quantified in mouse trabecular bone. It was found that parameters such as trabecular thickness and connectivity density are strongly affected by scan voxel size, while other parameters such as trabecular number and trabecular separation are less dependent on voxel size. Comparisons were also made between qualitative selection of segmentation threshold and quantitative selection based on image histogram. It was found that both segmentation methods yielded comparable results for scans with small voxel sizes, but diverged for some outcomes at larger voxel sizes.

Micro-computed tomography scan voxel size strongly affected several trabecular bone parameters that are commonly reported in small animal studies. In particular, trabecular thickness, connectivity density, and bone tissue mineral density were strongly dependent on scanning voxel size, regardless of the segmentation method utilized. In contrast, trabecular number, trabecular separation, and apparent bone mineral density were not strongly dependent on scan resolution for voxel sizes in the range of 6–20 μm. Scanning with a voxel size of 30 μm yielded predictably poor results, since this is approximately the dimension of a mouse trabecula. Generally, as the ratio of object size to voxel size decreases, the measurement error increases. Ideally, the ratio should be as high as possible for accurate morphologic measurements. The local solution accuracy of μCT-based finite element models is also influenced by voxel size, and a minimum discretization of three to four elements across the thickness of individual trabeculae is recommended to minimize numerical errors (Guldberg et al., 1998).

Comparing the two segmentation methods used in this study, it was found that the Qualitatively Selected segmentation method was more effective for estimating BV/TV and Tb.Th across voxel sizes, while the Histogram segmentation methods was more effective at estimating Tb.N, Tb.Sp, and SMI. BV/TV is the primary outcome of most studies reporting trabecular bone parameters, and was not strongly affected by voxel size when the Qualitatively Selected segmentation method was used, although increasing voxel size was associated with increasing within-group standard deviation. This indicates that accurate BV/TV values can be obtained for an array of voxel sizes if the segmentation threshold is carefully selected to be as physiologically representative as possible. Both segmentation methods yielded similar results for connectivity density, bone tissue mineral density, and apparent bone mineral density (which is not dependent on segmentation method). Results for connectivity density and bone tissue mineral density were highly dependent on scanning voxel size, even when comparing 6 μm voxel size to 10 μm voxel size. This lack of convergence even at small voxel size may make these outcomes difficult to compare across studies or using different scanners.

Qualitative selection of segmentation threshold and quantitative determination of threshold according to image histogram yielded similar thresholds for scans with small voxel sizes (high resolution), but correlated poorly for large voxel sizes. This may be due to the fact that the histogram peak for voxels designated as “bone” differed considerably for scans from 6 um to 30 μm voxel size (from native threshold value 480 at 6 μm to 270 at 30 μm), while the histogram peak for “non-bone” differed to a much less extent (from native threshold value 115 at 6 μm to 140 at 30 μm). Since the higher resolution scans provided greater separation between the two peaks, they were more successful at obtaining physiologically representative segmentation of trabecular bone. According to the ASBMR Guidelines for Assessment of Bone Microstructure in Rodents Using Micro-Computed Tomography, it is essential to compare 2D images from the original and segmented images to ensure that the extracted bone is a good representation of the actual structure (Bouxsein et al., 2010). In this regard, quantitative determination of segmentation threshold based on image histogram was successful for scans with small voxel sizes, but failed the “eye test” for scans with larger voxels. For scans with structure thickness:voxel size ratios that approach 2:1 (approximately 15 μm voxel size for this study) it may be more effective to select a segmentation threshold based on qualitative assessment of images.

Previous studies of CT or μCT resolution have also observed dependence of trabecular bone parameters on voxel size (Kim et al., 2004, Sode et al., 2008, Isaksson et al., 2011, Peyrin et al., 1998, Kothari et al., 1998). Isaksson et al. (Isaksson et al., 2011) used μCT to investigate whether image resolution affects bone structural parameters differently in healthy normal and osteoporotic trabecular bone. They found that with increasing image voxel size, the originally detected differences between normal and osteoporotic groups diminished, suggesting that structural differences between osteoporotic and normal trabecular bone may not be reliably detected with clinical CT scanners providing image voxel sizes above 100 μm. Kim et al. (2004) used three different scanning and reconstruction voxel sizes representing high resolution voxel size (21 μm), commonly used intermediate voxel size (50 μm), and voxel size applicable to scans of whole human vertebral bodies (110 μm) in order to examine the effect of voxel size on stereological measures for human cancellous bone. They found that the error in stereological parameters calculated using large voxel sizes compared to high resolution voxel size ranged from 0.1% to 102%. Peyrin et al. (1998) examined a series of vertebrae samples from healthy females of different ages (33 to 90) with voxel sizes of 14, 6.7 and 1.4 μm. They concluded that voxel sizes as large as 14 μm provide a reasonably good parameterization of trabecular architecture. Altogether, these findings are in agreement with the findings of this study, and suggest that large μCT voxel sizes may not provide an accurate description of trabecular bone structure.

To our knowledge, this study is the first to show the effect of μCT voxel size and segmentation method on trabecular bone structural outcomes specific to mouse studies. In comparing two commonly used segmentation methods across scan voxel sizes from 6–30 μm, this study was able to show the effect of voxel size on the most commonly reported trabecular bone outcomes. However, this study had several important limitations. First, our analysis did not utilize more advanced methods for segmentation of trabecular bone (Rajon et al., 2006, Burghardt et al., 2007, Buie et al., 2007, Scherf and Tilgner, 2009, Liu et al., 2010, Hojjat et al., 2010, Gomez et al., 2013, Waarsing et al., 2004). It is possible that these advanced segmentation methods would provide a more robust estimation of trabecular bone parameters at larger voxel sizes. Second, trabecular bone was analyzed at only one skeletal site in mice; it is unclear how generalizable the findings of this study are to other trabecular bone sites. A more thorough analysis could analyze multiple skeletal sites in mice, or other small animal models such as rats. Similarly, this analysis did not include trabecular bone from animals with low or high bone mass phenotypes. It is possible that the effect of voxel size on trabecular bone outcomes is different for regions with high or low trabecular bone volume fractions. Finally, this study utilized only one μCT scanner, with the associated analysis software provided by the manufacturer. It is unclear how the findings of this study would translate to other scanners or analysis software. Theoretically, these findings would be translatable to μCT scanners and analysis software from other manufacturers; however, this remains to be shown.

## 5 Conclusions

This study found that many commonly reported trabecular bone structure outcomes are significantly affected by μCT scanning voxel size and the global segmentation method used to delineate bone from non-bone. Based on these data, it is recommended that high-resolution scans be used whenever possible to provide the most accurate estimation of trabecular bone microstructure. If high-resolution scans are not possible, the limitations of accurately determining trabecular bone outcomes should be considered when making conclusions about inter-group variance or between-group differences.

## Conflict of interest

The author has no conflicts of interest to disclose.
